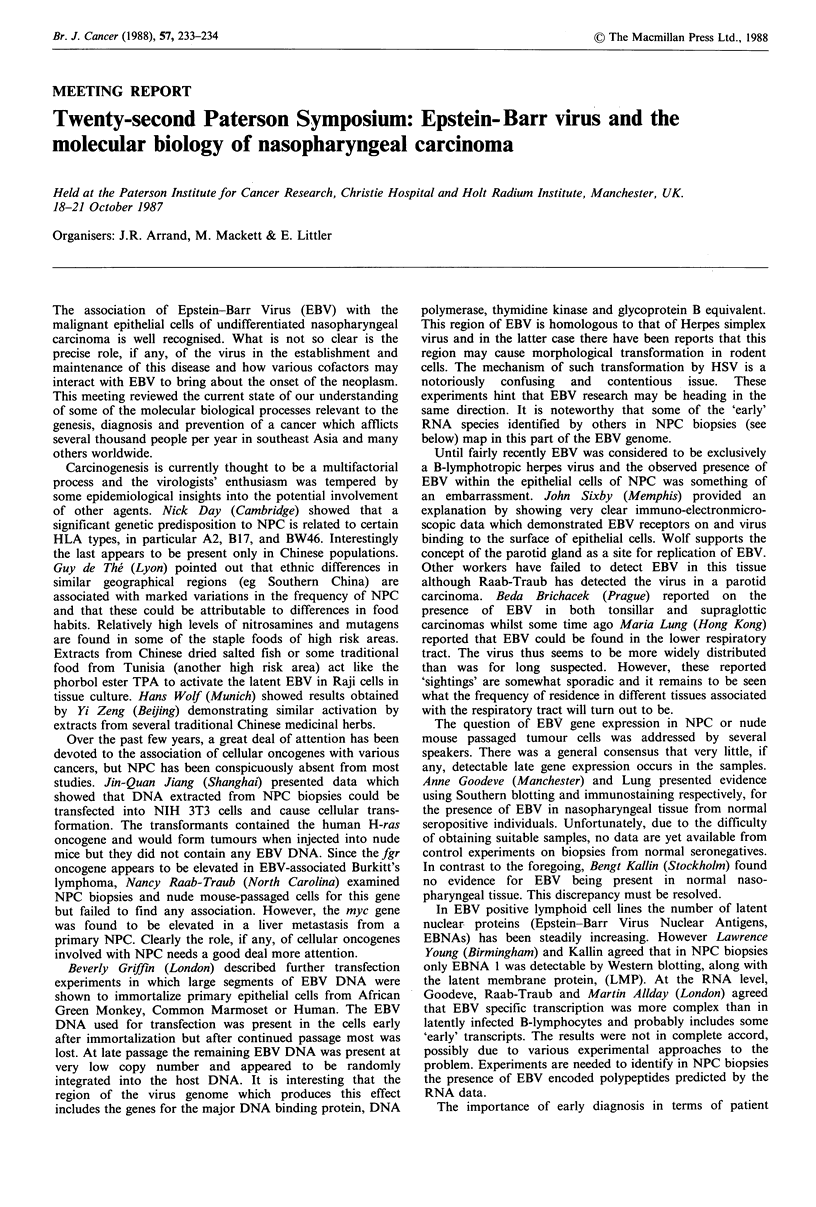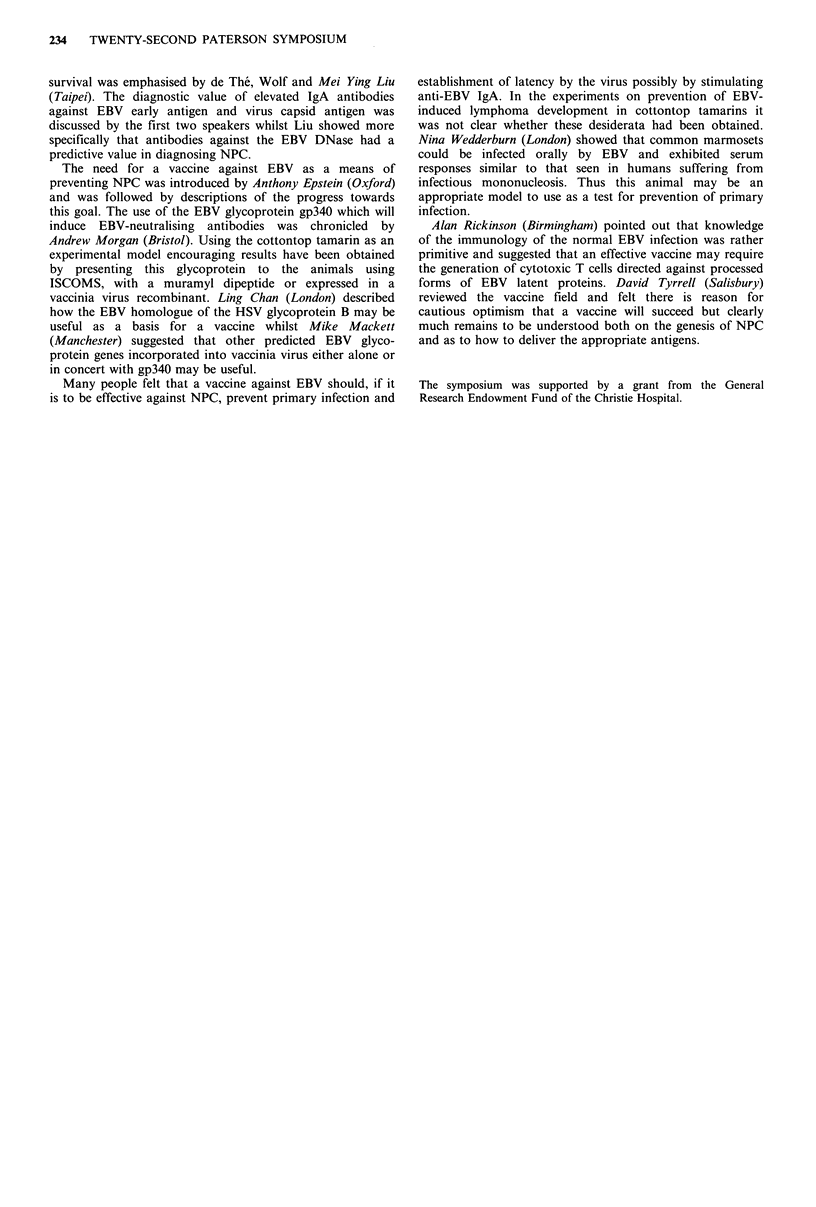# Twenty-second Paterson Symposium: Epstein-Barr Virus and the Molecular Biology of Nasopharyngeal Carcinoma

**Published:** 1988-02

**Authors:** 


*Br. J. Cancer* (1988), **57**, 233–234 © The Macmillan Press Ltd., 1988

MEETING REPORT

### Twenty-second Paterson Symposium: Epstein-Barr virus and the molecular biology of nasopharyngeal carcinoma

*Held at the Paterson Institute for Cancer Research, Christie Hospital and Holt Radium Institute, Manchester, UK. 18–21 October 1987*

Organisers: J.R. Arrand, M. Mackett & E. Littler

The association of Epstein–Barr Virus (EBV) with the malignant epithelial cells of undifferentiated nasopharyngeal carcinoma is well recognised. What is not so clear is the precise role, if any, of the virus in the establishment and maintenance of this disease and how various cofactors may interact with EBV to bring about the onset of the neoplasm. This meeting reviewed the current state of our understanding of some of the molecular biological processes relevant to the genesis, diagnosis and prevention of a cancer which afflicts several thousand people per year in southeast Asia and many others worldwide.

Carcinogenesis is currently thought to be a multifactorial process and the virologists' enthusiasm was tempered by some epidemiological insights into the potential involvement of other agents. *Nick Day* (*Cambridge*) showed that a significant genetic predisposition to NPC is related to certain HLA types, in particular A2, B17, and BW46. Interestingly the last appears to be present only in Chinese populations. *Guy de Thé* (*Lyon*) pointed out that ethnic differences in similar geographical regions (eg Southern China) are associated with marked variations in the frequency of NPC and that these could be attributable to differences in food habits. Relatively high levels of nitrosamines and mutagens are found in some of the staple foods of high risk areas. Extracts from Chinese dried salted fish or some traditional food from Tunisia (another high risk area) act like the phorbol ester TPA to activate the latent EBV in Raji cells in tissue culture. *Hans Wolf* (*Munich*) showed results obtained by *Yi Zeng* (*Beijing*) demonstrating similar activation by extracts from several traditional Chinese medicinal herbs.

Over the past few years, a great deal of attention has been devoted to the association of cellular oncogenes with various cancers, but NPC has been conspicuously absent from most studies. *Jin-Quan Jiang* (*Shanghai*) presented data which showed that DNA extracted from NPC biopsies could be transfected into NIH 3T3 cells and cause cellular transformation. The transformants contained the human H-*ras* oncogene and would form tumours when injected into nude mice but they did not contain any EBV DNA. Since the *fgr* oncogene appears to be elevated in EBV-associated Burkitt's lymphoma, *Nancy Raab-Traub* (*North Carolina*) examined NPC biopsies and nude mouse-passaged cells for this gene but failed to find any association. However, the *myc* gene was found to be elevated in a liver metastasis from a primary NPC. Clearly the role, if any, of cellular oncogenes involved with NPC needs a good deal more attention.

*Beverly Griffin* (*London*) described further transfection experiments in which large segments of EBV DNA were shown to immortalize primary epithelial cells from African Green Monkey, Common Marmoset or Human. The EBV DNA used for transfection was present in the cells early after immortalization but after continued passage most was lost. At late passage the remaining EBV DNA was present at very low copy number and appeared to be randomly integrated into the host DNA. It is interesting that the region of the virus genome which produces this effect includes the genes for the major DNA binding protein, DNA polymerase, thymidine kinase and glycoprotein B equivalent. This region of EBV is homologous to that of Herpes simplex virus and in the latter case there have been reports that this region may cause morphological transformation in rodent cells. The mechanism of such transformation by HSV is a notoriously confusing and contentious issue. These experiments hint that EBV research may be heading in the same direction. It is noteworthy that some of the 'early' RNA species identified by others in NPC biopsies (see below) map in this part of the EBV genome.

Until fairly recently EBV was considered to be exclusively a B-lymphotropic herpes virus and the observed presence of EBV within the epithelial cells of NPC was something of an embarrassment. *John Sixby* (*Memphis*) provided an explanation by showing very clear immuno-electronmicroscopic data which demonstrated EBV receptors on and virus binding to the surface of epithelial cells. Wolf supports the concept of the parotid gland as a site for replication of EBV. Other workers have failed to detect EBV in this tissue although Raab-Traub has detected the virus in a parotid carcinoma. *Beda Brichacek* (*Prague*) reported on the presence of EBV in both tonsillar and supraglottic carcinomas whilst some time ago *Maria Lung* (*Hong Kong*) reported that EBV could be found in the lower respiratory tract. The virus thus seems to be more widely distributed than was for long suspected. However, these reported 'sightings' are somewhat sporadic and it remains to be seen what the frequency of residence in different tissues associated with the respiratory tract will turn out to be.

The question of EBV gene expression in NPC or nude mouse passaged tumour cells was addressed by several speakers. There was a general consensus that very little, if any, detectable late gene expression occurs in the samples. *Anne Goodeve* (*Manchester*) and Lung presented evidence using Southern blotting and immunostaining respectively, for the presence of EBV in nasopharyngeal tissue from normal seropositive individuals. Unfortunately, due to the difficulty of obtaining suitable samples, no data are yet available from control experiments on biopsies from normal seronegatives. In contrast to the foregoing, *Bengt Kallin* (*Stockholm*) found no evidence for EBV being present in normal nasopharyngeal tissue. This discrepancy must be resolved.

In EBV positive lymphoid cell lines the number of latent nuclear proteins (Epstein–Barr Virus Nuclear Antigens, EBNAs) has been steadily increasing. However *Lawrence Young* (*Birmingham*) and Kallin agreed that in NPC biopsies only EBNA 1 was detectable by Western blotting, along with the latent membrane protein, (LMP). At the RNA level, Goodeve, Raab-Traub and *Martin Allday* (*London*) agreed that EBV specific transcription was more complex than in latently infected B-lymphocytes and probably includes some 'early' transcripts. The results were not in complete accord, possibly due to various experimental approaches to the problem. Experiments are needed to identify in NPC biopsies the presence of EBV encoded polypeptides predicted by the RNA data.

The importance of early diagnosis in terms of patient survival was emphasised by de Thé, Wolf and *Mei Ying Liu* (*Taipei*). The diagnostic value of elevated IgA antibodies against EBV early antigen and virus capsid antigen was discussed by the first two speakers whilst Liu showed more specifically that antibodies against the EBV DNase had a predictive value in diagnosing NPC.

The need for a vaccine against EBV as a means of preventing NPC was introduced by *Anthony Epstein* (*Oxford*) and was followed by descriptions of the progress towards this goal. The use of the EBV glycoprotein gp340 which will induce EBV-neutralising antibodies was chronicled by *Andrew Morgan* (*Bristol*). Using the cottontop tamarin as an experimental model encouraging results have been obtained by presenting this glycoprotein to the animals using ISCOMS, with a muramyl dipeptide or expressed in a vaccinia virus recombinant. *Ling Chan* (*London*) described how the EBV homologue of the HSV glycoprotein B may be useful as a basis for a vaccine whilst *Mike Mackett* (*Manchester*) suggested that other predicted EBV glycoprotein genes incorporated into vaccinia virus either alone or in concert with gp340 may be useful.

Many people felt that a vaccine against EBV should, if it is to be effective against NPC, prevent primary infection and establishment of latency by the virus possibly by stimulating anti-EBV IgA. In the experiments on prevention of EBV-induced lymphoma development in cottontop tamarins it was not clear whether these desiderata had been obtained. *Nina Wedderburn* (*London*) showed that common marmosets could be infected orally by EBV and exhibited serum responses similar to that seen in humans suffering from infectious mononucleosis. Thus this animal may be an appropriate model to use as a test for prevention of primary infection.

*Alan Rickinson* (*Birmingham*) pointed out that knowledge of the immunology of the normal EBV infection was rather primitive and suggested that an effective vaccine may require the generation of cytotoxic T cells directed against processed forms of EBV latent proteins. *David Tyrrell* (*Salisbury*) reviewed the vaccine field and felt there is reason for cautious optimism that a vaccine will succeed but clearly much remains to be understood both on the genesis of NPC and as to how to deliver the appropriate antigens.

The symposium was supported by a grant from the General Research Endowment Fund of the Christie Hospital.